# High‐Throughput Quantitative Chemical Shift‐Encoded MRI of the Liver

**DOI:** 10.1002/jmri.70429

**Published:** 2026-07-09

**Authors:** Garrett C. Fullerton, Jiayi Tang, Jitka Starekova, Julius F. Heidenreich, Ali Pirasteh, David T. Harris, Daiki Tamada, Collin J. Buelo, Dan W. Rettmann, Diego Hernando, Scott B. Reeder

**Affiliations:** ^1^ Department of Radiology University of Wisconsin‐Madison Madison Wisconsin USA; ^2^ Department of Medical Physics University of Wisconsin‐Madison Madison Wisconsin USA; ^3^ GE HealthCare Waukesha Wisconsin USA; ^4^ Department of Biomedical Engineering University of Wisconsin‐Madison Madison Wisconsin USA; ^5^ Department of Electrical and Computer Engineering University of Wisconsin‐Madison Madison Wisconsin USA; ^6^ Department of Medicine University of Wisconsin‐Madison Madison Wisconsin USA; ^7^ Department of Emergency Medicine University of Wisconsin‐Madison Madison Wisconsin USA

**Keywords:** automated image prescription, chemical shift‐encoded MRI, free‐breathing MRI, high‐throughput MRI, MRI workflow, proton density fat fraction

## Abstract

**Background:**

Access to MRI is limited by lengthy exam times and inefficient utilization. Focused protocols can reduce exam times, but workflow variability and inefficient room turnaround contribute to conservative scheduling with long exam slots.

**Purpose:**

To develop and evaluate a high‐throughput clinical MRI suite architecture and workflow, using an AI‐prescribed free‐breathing chemical shift‐encoded (CSE) MRI exam to quantify liver proton density fat fraction (PDFF) in under 5 min of total MRI room time.

**Study Type:**

Prospective.

**Subjects:**

24 healthy volunteers in two cohorts: 12 research staff (7 women/5 men; age 26.8 ± 5.8 years) and 12 community volunteers (6 women/6 men; age 41.3 ± 13.5 years).

**Field Strength/Sequence:**

1.5 T; free‐breathing 2D multi‐echo gradient echo CSE‐MRI.

**Assessment:**

Each participant underwent three nonconsecutive CSE‐MRI exams in a continuously queued workflow to characterize timing and PDFF repeatability. Workflow intervals were recorded from timestamped video review and image metadata. Staff cohort exams included two CSE‐MRI acquisitions to assess within‐exam repeatability, while community cohort exams included one to simulate clinical practice. Three radiologists (8/13/14 years of experience) independently evaluated AI‐automated prescriptions for complete liver coverage and rated CSE‐MRI image quality (five‐point Likert scale).

**Statistical Tests:**

Student's *t*‐tests; Gwet's AC2; repeatability coefficients (RCs) with bootstrap 95% confidence intervals; Bland–Altman analysis. *p* < 0.05 was significant.

**Results:**

Diagnostic image quality was achieved in all 72 exams (median PDFF Likert score 5/5, inter‐rater AC2 ≥ 0.86). Total MRI room times averaged 4:09 ± 0:14 min (staff) and 3:35 ± 0:34 min (community). Turnaround times averaged under 2 min, enabling throughput of 16.1 exams per hour in the community cohort. Automated prescription achieved complete liver coverage in all exams. PDFF RCs were 0.78% (staff within‐exam), 0.99% (staff between‐exam), and 1.21% (community between‐exam) absolute PDFF.

**Data Conclusion:**

The proposed high‐throughput MRI workflow achieved over 16 exams per hour with highly repeatable liver fat quantification, demonstrating a framework for improving MRI utilization and access.

**Evidence Level:**

1.

**Stage of Technical Efficacy:**

2.

## Plain Language Summary

Liver disease affects over two billion people globally, and magnetic resonance imaging (MRI) can detect liver fat buildup that signals early disease. Liver MRI exams frequently take 30–60 min per patient, limiting access. This study tested a faster workflow combining three innovations: a redesigned MRI suite with movable tables that enable simultaneous patient preparation and imaging; software that automatically positions imaging volumes over the liver; and an imaging method that does not require breath‐holding, which slows conventional liver MRI. With this approach, over 16 patients could be imaged per hour while keeping fat measurements accurate and reliable.

## 1 Introduction

MRI is an important modality for the diagnosis, staging, and treatment monitoring of numerous diseases. Despite its clinical value, MRI has historically required lengthy exams, often exceeding one hour, contributing to patient discomfort and limiting access, as demand for imaging continues to grow [1, 2]. MRI also poses financial challenges for institutions, with high capital costs [3] and reimbursement that has fallen dramatically in recent years [4]. These factors have driven research and adoption of focused MRI protocols that use fewer sequences to efficiently address specific clinical questions [5, 6]. However, with conventional single‐door, fixed‐table MRI suites, room turnaround between patients can require 10 min or more [7]. Focused protocols increasingly require only a few minutes of imaging [8], such that exam slot durations are increasingly dominated by room turnaround.

Exam slots are often set to accommodate an upper percentile of exam times (e.g., 80th percentile), ensuring that most exams are completed within the scheduled window [9]. Timing variability often arises from patient‐dependent factors, such as breath‐holding, and from repeated acquisitions due to motion artifacts or other image quality issues [10]. Variability in both exam time and room turnaround time leads to conservative scheduling and poor utilization of MRI resources [11]. Thus, MRI utilization and patient access are increasingly limited by workflow rather than image acquisition time, particularly for focused MRI protocols [12, 13].

One validated application of focused MRI is chemical shift‐encoded MRI (CSE‐MRI) to quantify proton density fat fraction (PDFF), a well‐established biomarker of liver fat content [14]. PDFF is regarded as the most accurate and precise biomarker for detecting, grading, and monitoring metabolic dysfunction‐associated steatotic liver disease (MASLD) [15]. MASLD is increasingly prevalent, affecting over 100 million Americans and over two billion people worldwide [16]. The recent approval of effective therapies such as resmetirom [17] and semaglutide [18] to treat MASLD is expected to increase the need for noninvasive diagnosis and treatment monitoring. For these reasons, demand for PDFF is already surging, reinforcing the importance of high‐throughput workflow strategies [19, 20].

In light of these evolving circumstances, many clinical practices are actively pursuing strategies that improve clinical MRI workflow and throughput [12]. One such strategy uses a two‐door MRI architecture with two mobile detachable tables, facilitating preparation of patients in an adjacent room while the preceding patient undergoes imaging [7]. In this design, patients are transported directly to and from the MRI system without setting foot in the MRI room, minimizing transition delays and variability. AI‐based automatic image prescription can further improve throughput by streamlining technologist workflow [21]. Additionally, motion‐insensitive CSE‐MRI methods can be used to enable free‐breathing exams, eliminating workflow complications associated with breath‐holding [22, 23]. Combining these approaches could enable high‐quality, repeatable liver fat quantification in under 5 min of total MRI exam time. Therefore, the purpose of this work was to demonstrate the feasibility and performance of a high‐throughput clinical MRI suite architecture and workflow using an AI‐assisted, free‐breathing CSE‐MRI exam to quantify liver fat.

## 2 Materials and Methods

This Health Insurance Portability and Accountability Act (HIPAA)–compliant prospective study was designed to evaluate a high‐throughput MRI suite and workflow through two substudies, each performed in a separate cohort. The study was approved by the institutional review board (IRB), and written informed consent was obtained from all participants.

The first cohort (research staff volunteers) provided initial feasibility testing with within‐exam repeatability assessment. The second cohort (community volunteers) was intended to represent a clinically relevant population. The workflow incorporated three key technologies: (1) a two‐door MRI suite with two detachable tables enabling rapid room turnaround, (2) AI‐based automatic image prescription (“autoprescription”), and (3) motion‐insensitive 2D CSE‐MRI for free‐breathing liver fat quantification.

### 2.1 MRI Suite Design

Inspired by Recht et al. [7], a “smart suite” was designed and constructed at UW Health Eastpark Medical Center, opening for clinical use in October 2024 (Figure 1A). The smart suite includes two doors, a separate preparation (“prep”) room where all coils are stored, two mobile tables, and an MRI system oriented at 45° to facilitate table movement. The workflow (Figure 1B) begins with positioning the patient and coils on a table in the prep room while the preceding exam occurs. The table is then wheeled into the MRI room through one door and docked, and the patient is landmarked and advanced into the bore. Immediately after the exam is complete, the table is undocked and wheeled out through a second door into the control room. At the same time, the next patient is transported into the MRI room through the first door on another table. The first patient is transported from the control room into the prep room, where they get off the table. Linens are then changed, and table and coil surfaces are cleaned in preparation for the next patient. Notably, in this workflow, patients never set foot on the MRI room floor, minimizing variability that would otherwise arise from differences in patient mobility.

**FIGURE 1:**
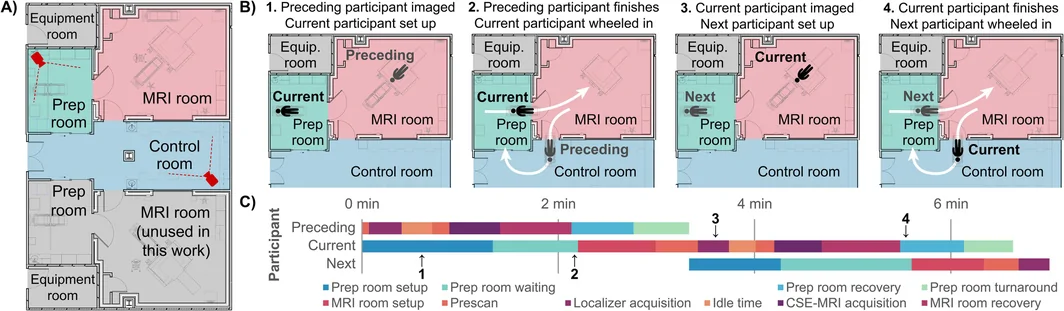
Illustration of (A) the suite architecture, (B) workflow, and (C) timing for focused CSE‐MRI exams in the two‐door MRI smart suite. Shown in (A) is an architectural diagram of the twin two‐door MRI smart suites installed at the study site. In this work, only one of the two MRI rooms was used. Positions and fields of view of video cameras used to record timings for workflow intervals (see Table 1) are depicted in red. Shown in (B) is the journey of one participant (“current participant”) in the smart suite. Notably, no participants ever set foot on the MRI room floor. Shown in (C) is a timeline with representative durations for each stage of the workflow. Numbered arrows correspond with illustrations of workflow in (B). The overlapping of workflow stages between different participants is also illustrated. CSE‐MRI, chemical shift‐encoded MRI.

Each substudy was conducted by five staff members (1–9 years of MRI experience): one technologist at the MRI console and four other staff divided into two teams, with each team dedicated to a single mobile table. These teams handled participant preparation and transport, enabling continuous parallel operation (Figure 1C). While fewer staff may be sufficient in routine clinical practice, this configuration was chosen to ensure that workflow was not limited by staffing.

### 2.2 Study Participants

Participants were enrolled in two cohorts between September and November 2025. The staff cohort (university research staff volunteers familiar with the MRI environment) provided an initial feasibility assessment. The community cohort was recruited from an IRB‐approved general‐public volunteer database, targeting diverse age, sex, and body mass index (BMI) to evaluate performance in a population more representative of patients at risk for MASLD. This cohort also enabled assessment of workflow in participants unfamiliar with the MRI environment.

Prior to each substudy, all participants completed MRI safety screening, and those with routine contraindications to MRI were excluded. Participants changed into MRI‐safe clothing in dedicated changing rooms outside the MRI suite and did not change clothes between exams. Both substudies were performed in the evening following the daily clinical schedule. Food was provided between exams, and participants were not required to fast or limit food intake.

### 2.3 Imaging Protocol

Participants were imaged in a fixed, cyclic order repeated three times, such that each participant underwent three CSE‐MRI exams. In the staff cohort, two identical CSE‐MRI acquisitions were performed per exam to evaluate within‐exam repeatability. In the community cohort, only one CSE‐MRI acquisition was performed per exam to more accurately simulate a clinical protocol, as repeated acquisitions are not typically performed in practice.

All imaging was performed on a 1.5 T clinical MRI system (SIGNA Artist, GE HealthCare, Waukesha, WI) using a 30‐channel flexible anterior coil array and 40‐channel posterior embedded table coil array.

Free‐breathing single‐shot fast spin echo (SSFSE) three‐plane localizers were acquired using standard clinical parameters (10 coronal, 10 sagittal, and 5 axial slices; 8 mm thickness, no gap). AI‐assisted autoprescription was implemented using a deep‐learning network trained to identify liver position from three‐plane localizers [21, 24]. Network outputs were converted to graphical prescriptions via a vendor‐provided prototype autoprescription platform (GE HealthCare).

CSE‐MRI was performed using a motion‐insensitive 2D flip angle‐modulated (FAM) multi‐echo spoiled gradient‐echo method acquired during free‐breathing. FAM CSE‐MRI uses sequential 2D encoding with a sub‐second temporal aperture per slice that is insensitive to respiratory motion [25]. Further, FAM uses centric phase encoding with optimized flip angles modulated across phase‐encoding lines to improve SNR while minimizing k‐space filtering effects and T1‐related bias in PDFF estimation [26, 27].

CSE‐MRI data were acquired with 6 monopolar echoes (TE_1_ = 1.40 ms, ΔTE = 1.82 ms) using the following parameters: 40 × 40 cm^2^ FOV, 144 × 144 matrix (2.8 × 2.8 mm^2^ in‐plane resolution), 2× auto‐calibrated parallel imaging acceleration in the phase‐encoding direction [26, 28], TR = 12.4 ms, 8 mm slice thickness, and no slice gap. These parameters were taken from FAM CSE‐MRI protocols in use clinically at UW Health and previously evaluated by Heidenreich et al. [29]. The acquisition matrix is compliant with Quantitative Imaging Biomarkers Alliance recommendations for MRI‐based liver PDFF mapping [30]. A variable number of axial slices were prescribed by the autoprescription platform, targeting complete liver coverage. Prescription volumes were expanded by 20 mm in the superior/inferior directions to accommodate potential liver motion between the free‐breathing localizer and CSE‐MRI acquisitions [31]. Confounder‐corrected PDFF and R2* maps were simultaneously reconstructed using vendor‐provided software that accounts for R2*‐decay and multipeak spectral modeling of fat [32, 33]. While PDFF was the primary outcome of this study, R2* maps provide a quantitative biomarker of tissue iron content and were evaluated as a complementary measure.

### 2.4 Exam Timing

Video recordings of all exams provided timing data for workflow transitions (e.g., prep and MRI room entry/exit). Participants' faces were blurred [34] for privacy and IRB compliance. Image metadata provided additional acquisition start/stop timing information. Workflow intervals and derived metrics, adapted from Chu et al. [35] and defined in Table **1**, were used to collate all timing data, which served as the reference for objective evaluation of the performance of the high‐throughput MRI exams.

**TABLE 1:** Definitions of workflow intervals and derived metrics used to characterize the high‐throughput MRI workflow. Intervals were timed using video recordings and image metadata. Imaging time and idle time are sub‐components of exam time.

Term	Description	Start	End	Data source
Workflow intervals				
Prep room setup	Participant positioning on table and coil placement	Prep room entry	Preparation complete	Video
Prep room waiting	Waiting for preceding exam to complete	Preparation complete	MRI room entry	Video
MRI room setup	Table docking, landmarking, and advancing participant into bore	MRI room entry	Localizer prescan start	Video, metadata
Exam time	Imaging time and idle time (see below)	Localizer prescan start	CSE‐MRI end	Metadata
Imaging time	Localizer and CSE‐MRI prescans and acquisitions	—	—	Metadata
Idle time	Autoprescription and waiting between acquisitions	—	—	Metadata
MRI room recovery	Participant withdrawal from bore and table undocking	CSE‐MRI end	MRI room exit	Metadata, video
Prep room recovery	Coil removal and participant dismount from table	MRI room exit	Prep room exit	Video
Prep room turnaround	Table cleaning, linen changing, coil cleaning, and preparation for next participant	Prep room exit	Next prep room entry	Video
MRI room turnaround	MRI room recovery of preceding participant and MRI room setup of next participant	Preceding exam end	Next exam start	Metadata
Derived metrics				
Visit time	Sum of prep room setup, prep room waiting, MRI room setup, exam time, MRI room recovery, and prep room recovery	—	—	—
MRI room time	Sum of MRI room setup, exam time, and MRI room recovery	—	—	—
MRI room usage	Ratio of exam time to MRI room time, expressed as a percentage	—	—	—

Abbreviation: CSE‐MRI, chemical shift‐encoded MRI.

### 2.5 Image Analysis

#### 2.5.1 Autoprescription Coverage

Three board‐certified radiologists (J.H., A.P., J.S.; 8, 13, and 14 years of experience) independently evaluated all PDFF and R2* volumetric datasets for complete liver coverage (yes/no). Minimal intersection of the extreme superior or inferior liver margin on the most cranial or caudal slice was considered complete coverage, as additional slices would not contain further liver tissue. An analyst (J.T., 4 years of experience in liver MRI) counted the number of slices not containing liver tissue at the superior and inferior ends of each acquisition to quantify excess coverage margin.

#### 2.5.2 Image Quality and Quantification

All three radiologists (J.H., A.P., J.S.) also independently rated overall image quality using a five‐point Likert scale previously described by Heidenreich et al. [29]. One radiologist (J.S.) analyzed each acquired volumetric dataset by placing elliptical regions of interest (ROI) in each of the nine Couinaud segments [36] using medical image viewer software (Horos v4.0.1, Horos Project, Annapolis, MD). ROIs were drawn to be as large as possible within each segment while avoiding the liver edge, large vessels, bile ducts, lesions, or obvious artifacts. For datasets of the same subject, ROIs were colocalized across acquisitions using anatomical landmarks. Mean PDFF and R2* were recorded for each segment, and whole‐liver PDFF and R2* were computed as the unweighted average across all nine segments.

### 2.6 Statistical Analysis

#### 2.6.1 Exam Timing

Workflow interval durations were summarized as mean ± standard deviation (SD) and 80th percentile for each cohort. The 80th percentile is a practical metric used for scheduling exam slots, and is needed to determine the minimum exam slot length to accommodate most patients [9]. Unpaired two‐tailed Student's *t*‐tests were used to compare interval durations between cohorts (*p* < 0.05 was significant).

#### 2.6.2 Autoprescription Coverage

The percentage of exams with complete liver coverage was calculated. The total number of slices prescribed and the number of slices without liver tissue at each acquisition's superior and inferior ends were summarized as mean ± SD.

#### 2.6.3 Image Quality and Quantification

Radiologist image quality ratings for PDFF and R2* maps were pooled across the three radiologists and reported as proportions assigned each Likert score in each cohort. Inter‐rater agreement was assessed using Gwet's AC2 with quadratic weighting for ordinal data [37], reported with 95% confidence intervals. Agreement was interpreted using established thresholds [38]: 0.0–0.2 slight, 0.2–0.4 fair, 0.4–0.6 moderate, 0.6–0.8 substantial, and 0.8–1.0 almost perfect agreement.

PDFF and R2* repeatability were assessed using the repeatability coefficient (RC), calculated as 2.77 times the within‐subject SD of whole‐liver PDFF or R2* [39]. For the staff cohort, within‐exam RC was calculated from paired acquisitions within each exam, and between‐exam RC from variance across the three exams. For the community cohort, only between‐exam RC was calculated. 95% confidence intervals were computed by bootstrap simulation (10,000 iterations). PDFF and R2* repeatability were also visualized using an extended Bland–Altman approach that generalizes beyond two repeated measurements [40, 41].

## 3 Results

Twenty‐four participants (13 women/11 men; mean age 26.8 ± 5.8 years [staff] and 41.3 ± 13.5 years [community]) were recruited across two cohorts (*n* = 12 each; Table 2). No participants were excluded (Figure 2). All 72 imaging exams were successfully completed, producing 108 volumetric PDFF datasets.

**TABLE 2:** Participant demographics, quantitative liver biomarker values, and image quality ratings for each cohort. Continuous variables are reported as mean ± SD (range). PDFF map and R2* map image quality was rated independently by three radiologists on a five‐point Likert scale and reported as pooled counts across all three raters. Dataset counts differ between cohorts as the staff cohort included two CSE‐MRI acquisitions per exam.

	Staff cohort (*n* = 12)	Community cohort (*n* = 12)
Age (years)	26.8 ± 5.8 (22–39)	41.3 ± 13.5 (22–60)
Sex		
Women	7 (58.3%)	6 (50.0%)
Men	5 (41.7%)	6 (50.0%)
BMI (kg/m^2^)	25.1 ± 5.3 (18.1–37.4)	30.9 ± 6.2 (23.7–40.3)
PDFF	72 datasets	36 datasets
Whole‐liver PDFF (%)	2.3 ± 3.0 (0.5–12.5)	5.7 ± 7.6 (0.6–29.0)
Image quality		
Rated 5 (excellent)	113 (52.3%)	55 (50.9%)
Rated 4 (very good)	87 (40.3%)	47 (43.5%)
Rated 3 (moderate)	16 (7.4%)	5 (4.6%)
Rated 2 (fair but diagnostic)	0 (0.0%)	1 (0.9%)
Rated 1 (nondiagnostic)	0 (0.0%)	0 (0.0%)
AC2 (95% CI)	0.86 (0.84–0.90)	0.88 (0.83–0.92)
R2*	72 datasets	36 datasets
Whole‐liver R2* (s^−1^)	32.0 ± 4.5 (23.9–43.8)	33.9 ± 2.9 (28.9–41.6)
Image quality		
Rated 5 (excellent)	82 (38.0%)	31 (28.7%)
Rated 4 (very good)	122 (56.5%)	59 (54.6%)
Rated 3 (moderate)	12 (5.6%)	17 (15.7%)
Rated 2 (fair but diagnostic)	0 (0.0%)	1 (0.9%)
Rated 1 (nondiagnostic)	0 (0.0%)	0 (0.0%)
AC2 (95% CI)	0.91 (0.88–0.93)	0.91 (0.87–0.94)

Abbreviations: BMI, Body mass index; CI, Confidence interval; CSE‐MRI, Chemical shift‐encoded MRI; PDFF, Proton density fat fraction.

**FIGURE 2:**
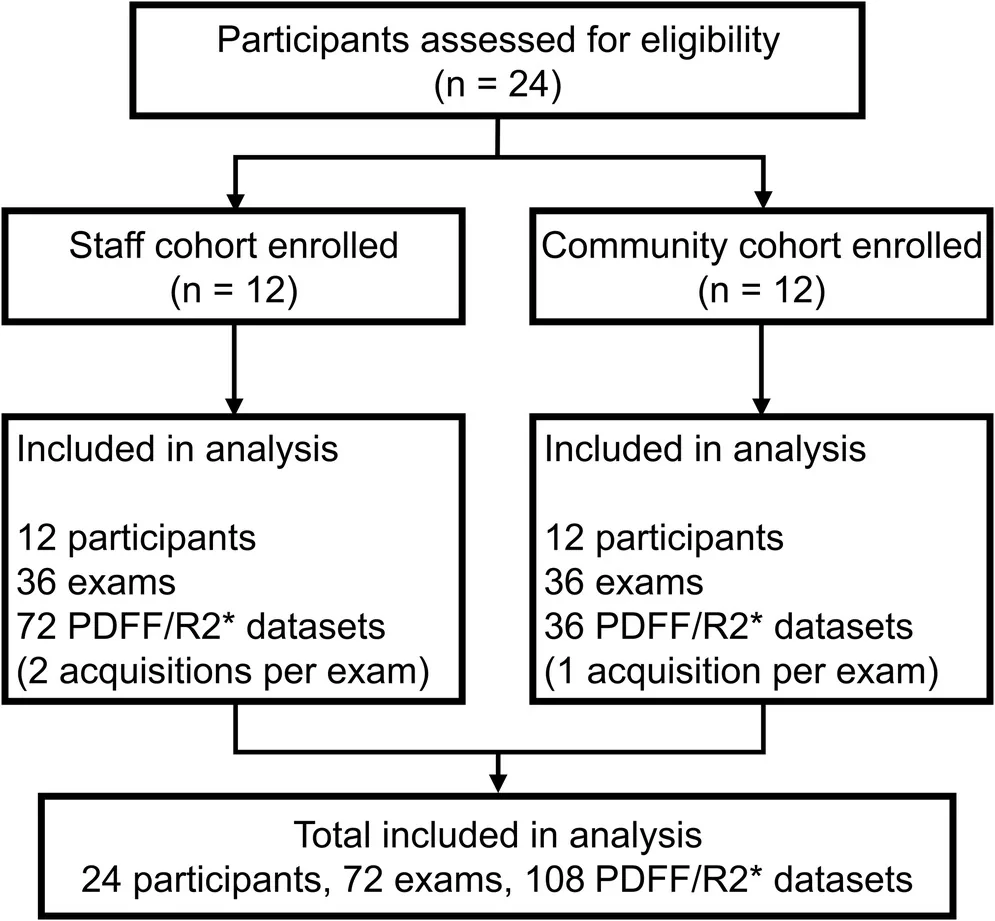
Flow diagram of participant enrollment and analysis. No participants were excluded from this study. PDFF, proton density fat fraction.

### 3.1 Exam Timing

All 36 staff cohort exams were completed in 2:34 h (14.0 exams/h; Figure 3A), and the community cohort exams were completed in 2:14 h (16.1 exams/h; Figure 3B). Detailed metrics for individual workflow intervals are presented in Table 3 and Figure 4.

**FIGURE 3:**
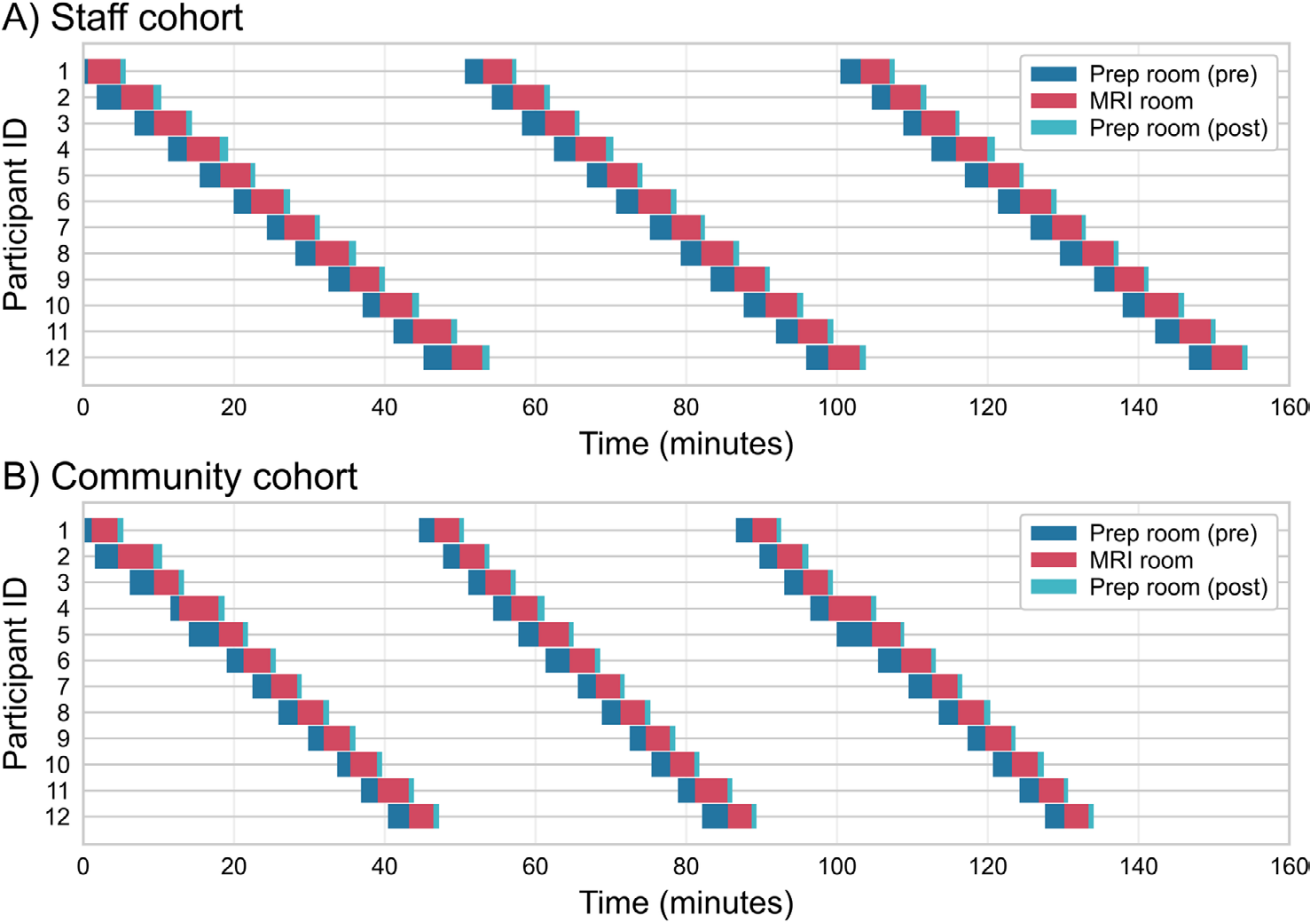
Rapid and consistent throughput with low variability between participants and exams was achieved for both cohorts, with total MRI room times of 4:09 ± 0:14 min (80th percentile 4:18 min) and 3:35 ± 0:34 (80th percentile 3:47 min) for the staff and community cohorts, respectively. All 36 exams were completed in 2:34 h (staff cohort; A) and 2:14 h (community cohort; B) demonstrating sustained high‐throughput operation. Each horizontal bar represents a single participant's progression through the workflow, with dark blue indicating prep room setup and waiting, red indicating MRI room occupancy, and light blue indicating post‐exam prep room recovery. The overlapping prep room and MRI room intervals illustrate continuous parallel workflow between participants.

**TABLE 3:** Both cohorts achieved high MRI exam throughput with low variability. Duration of workflow intervals and derived metrics are reported as mean (SD) and 80th percentile (80th %).

	Staff cohort		Community cohort		*p*
	Mean (SD)	80th %	Mean (SD)	80th %	
Prep room					
Setup	0:56 (0:11)	1:02	1:16 (0:17)	1:20	**< 0.001**
Waiting	1:49 (0:28)	2:05	1:16 (0:40)	1:30	**< 0.001**
Recovery	0:45 (0:09)	0:52	0:42 (0:07)	0:46	0.15
Total prep room time	3:30 (0:31)	3:43	3:15 (0:39)	3:40	0.07
MRI room					
Setup	0:53 (0:13)	0:57	0:51 (0:23)	0:51	0.74
Prescan	0:50 (0:04)	0:53	0:46 (0:15)	0:45	0.09
Localizer	0:20 (0:00)	0:20	0:20 (0:00)	0:20	**0.002** ^a^
CSE‐MRI	0:29 (0:02)	0:31	0:30 (0:03)	0:32	0.10
CSE‐MRI (repeat)^b^	0:29 (0:02)	0:31	—	—	**—**
Idle	0:23 (0:05)	0:25	0:20 (0:14)	0:19	0.18
Recovery	0:44 (0:03)	0:46	0:48 (0:13)	0:49	0.09
Total MRI room time	4:09 (0:14)	4:18	3:35 (0:34)	3:47	**< 0.001**
Turnaround					
Prep room turnaround	0:48 (0:17)	1:01	0:31 (0:15)	0:42	**< 0.001**
MRI room turnaround^c^	1:43 (0:13)	1:48	1:45 (0:26)	1:41	0.69
Summary					
Exam time (single CSE‐MRI)	1:44 (0:06)	1:48	1:56 (0:21)	1:56	**0.003**
Exam time	2:32 (0:07)	2:38	1:56 (0:21)	1:56	**< 0.001**
Visit time	7:39 (0:33)	8:00	6:50 (0:49)	7:13	**< 0.001**
MRI room usage	61.1% (3.4%)	63.3%	53.9% (6.0%)	56.3%	**< 0.001**

*Note:* Timing data are presented in minutes: seconds. *p*‐values reflect statistical comparisons between cohorts, and bold *p*‐values indicate significant differences (*p* < 0.05).

Abbreviation: CSE‐MRI, chemical shift‐encoded MRI.

^a^ Localizer acquisitions varied slightly in duration due to participant‐specific adjustments made automatically by the MRI system. All differences were less than 1 s, producing a statistically significant but clinically irrelevant difference.

^b^ A repeat CSE‐MRI acquisition was performed in the staff cohort only to assess within‐exam PDFF repeatability.

^c^ MRI room turnaround includes brief unoccupied intervals between consecutive participants.

**FIGURE 4:**
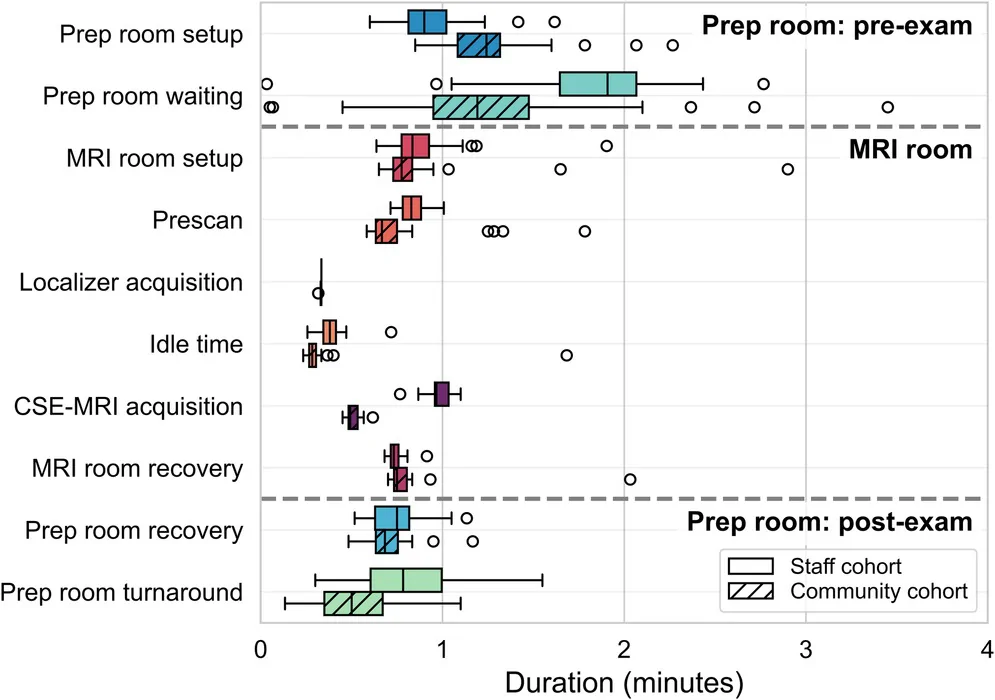
Summary of the workflow timing shows that short exam intervals with minimal variability were achieved for both cohorts. Box plots show the distribution of durations for each workflow interval for staff (solid boxes) and community (hatched boxes) cohorts. Individual outliers (circles) are shown for each interval. Prep room intervals (pre‐exam and post‐exam) are separated from MRI room intervals by dashed lines. The staff cohort completed two CSE‐MRI acquisitions per exam, resulting in longer CSE‐MRI acquisition times, while the community cohort completed one acquisition to simulate clinical practice. Both cohorts demonstrated rapid, reproducible performance across all intervals. CSE‐MRI, chemical shift‐encoded MRI.

The community cohort, which underwent what most closely represents a clinical protocol, achieved a mean total MRI room time of 3:35 ± 0:34 min (80th percentile: 3:47 min). Single‐acquisition exam time averaged under 2 min in both cohorts, with the staff cohort slightly but significantly shorter than the community cohort despite equivalent protocol length. MRI room turnaround times were consistent between cohorts (*p* = 0.69), averaging 1:43 and 1:45 min for the staff and community cohorts, respectively. Prep room setup times were significantly longer in the community cohort, while MRI room setup times were similar (*p* = 0.74).

Minor technical issues caused workflow interruptions in 7/72 (9.7%) exams. These included table docking/undocking failure (*n* = 2), autoprescription platform failures (*n* = 3, see below), prescan failure (*n* = 1), and participant activation of the emergency stop requiring acquisition restart (*n* = 1). These outliers are visible in Figure 4.

### 3.2 Autoprescription Coverage

Autoprescription functioned successfully in 69/72 exams without intervention. In two staff cohort exams, the vendor‐provided platform for converting network outputs to graphical prescriptions failed to initialize, requiring manual prescription. These failures were specific to the vendor‐provided platform and not the autoprescription network itself, which retrospective analysis confirmed would have produced acceptable prescriptions in both cases. In one community cohort exam, the platform required a restart before autoprescription was successfully performed.

All 72 exams achieved complete liver coverage as determined by all radiologists. Notably, autoprescription achieved accurate coverage even when participant landmarking varied between exams (see, e.g., Figure 5). The number of slices prescribed averaged 28.9 ± 2.5 (range: 22–36). Slices without liver tissue averaged 2.4 ± 1.4 (range: 0–8) and 2.7 ± 1.5 (range: 0–8) at the superior and inferior ends, respectively, corresponding to 19.2 ± 11.2 and 21.6 ± 12.0 mm of additional coverage and concordant with the intended 20 mm margin at each end. The additional slices acquired to accommodate these margins added 5.3 ± 2.1 s to each acquisition.

**FIGURE 5:**
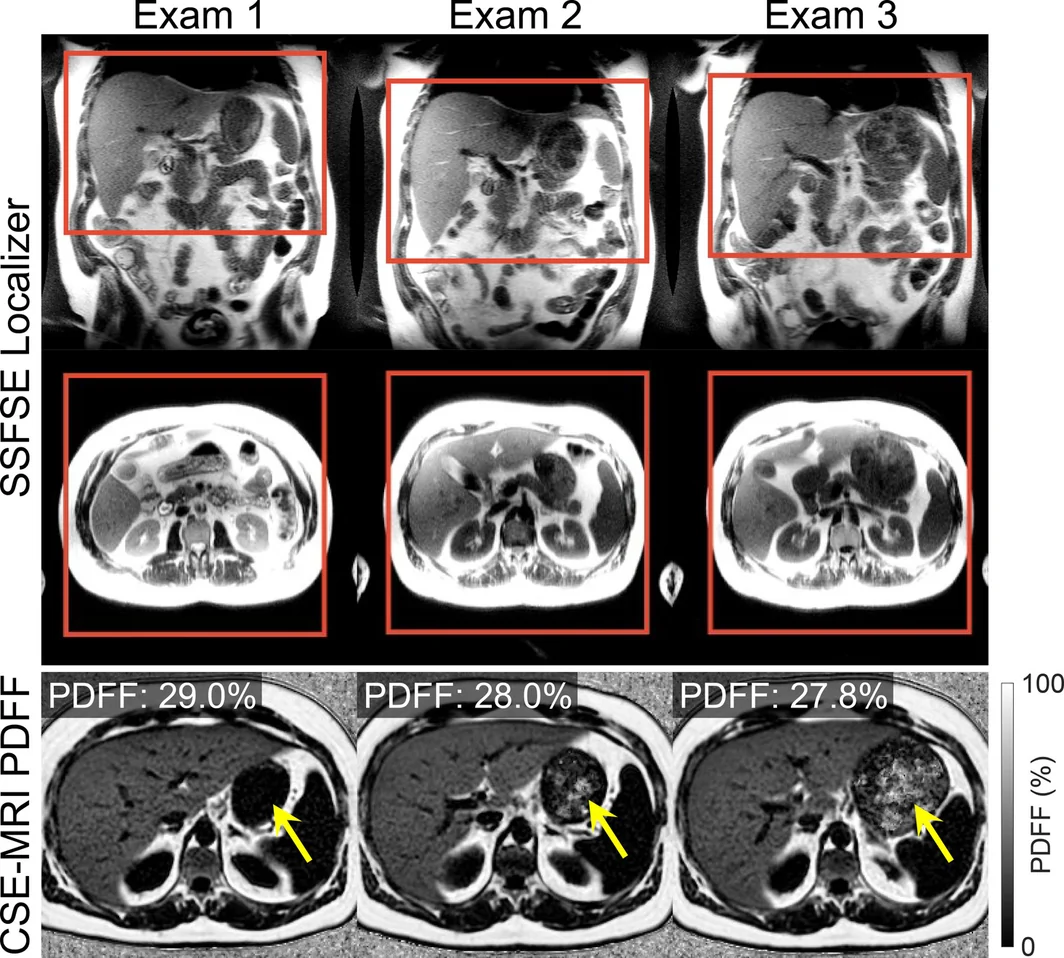
The proposed high‐throughput workflow produced consistent autoprescription results and high‐quality PDFF maps across all three sequential exams despite variations in initial positioning and localizer acquisitions. Representative SSFSE localizer images (coronal and axial) are shown, with autoprescription results shown as red boxes, demonstrating accurate liver coverage across all exams. Representative PDFF maps demonstrate very good to excellent image quality (rated 5 by two of three radiologists and 4 by the third) and are free of motion artifacts. Despite lower table‐based landmarking by the technologists in Exam 1, the autoprescription system successfully identified the liver. Mean whole‐liver PDFF values decreased slightly across exams (29.0%, 28.0%, 27.8%), likely reflecting increasing hydration as food and beverages were provided between exams (note progressively filling stomach, yellow arrow). Sagittal localizer images are not shown for brevity. CSE‐MRI, chemical shift‐encoded MRI; PDFF, proton density fat fraction; SSFSE, single‐shot fast spin echo.

### 3.3 Image Quality and Quantification

Representative SSFSE localizer images, autoprescription results, and PDFF maps are shown in Figure 5. Diagnostic image quality was achieved in all 72 exams for both PDFF and R2* maps. Pooled across the three radiologists, PDFF maps were rated as very good or excellent (Likert score 4 or 5) in 92.6% of staff and 94.4% of community acquisitions, with almost perfect inter‐rater agreement in both cohorts (AC2 = 0.86 [staff] and 0.88 [community]; Table 2). R2* maps showed similar quality, with 94.4% (staff) and 83.3% (community) rated 4 or 5, and AC2 = 0.91 in both cohorts (Table 2). Representative quantitative maps with various quality ratings are shown in Figure 6.

**FIGURE 6:**
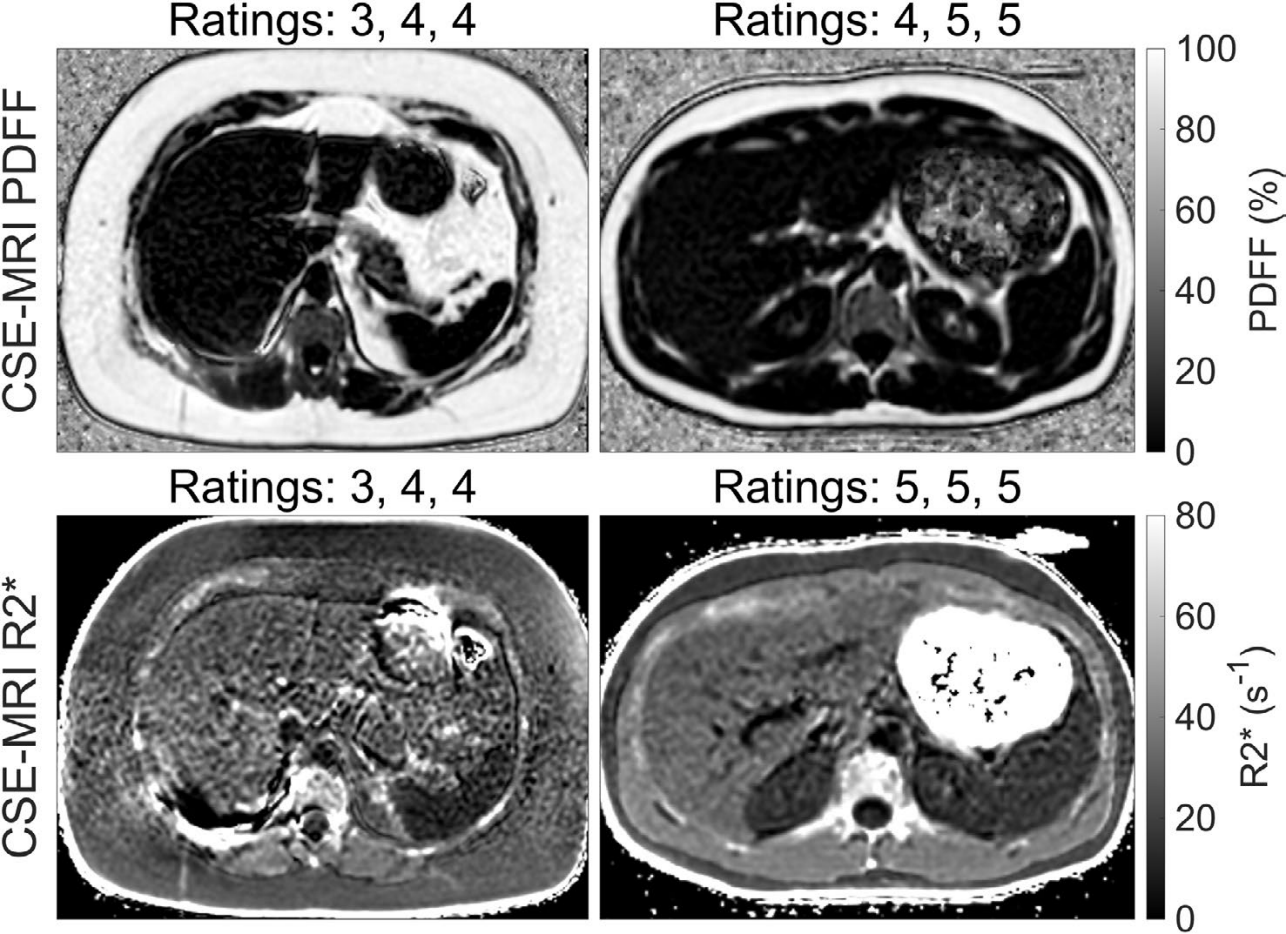
Example PDFF and R2* maps which received moderate and high image quality ratings from the three radiologist raters. PDFF, proton density fat fraction.

Mean whole‐liver PDFF was 2.3% ± 3.0% (range: 0.5%–12.5%) in the staff cohort and 5.7% ± 7.6% (range: 0.6%–29.0%) in the community cohort. Mean R2* was 32.0 ± 4.5 s^−1^ (range: 23.9–43.8 s^−1^) and 33.9 ± 2.9 s^−1^ (range: 28.9–41.6 s^−1^), respectively.

Quantitative repeatability in both cohorts is summarized in Figure 7. In the staff cohort, within‐exam RC for PDFF was 0.78% absolute PDFF (95% confidence interval [CI]: 0.61–0.96) and between‐exam RC was 0.99% absolute PDFF (95% CI: 0.74–1.28). Between‐exam RC in the community cohort was 1.21% absolute PDFF (95% CI: 0.87–1.49), comparable to the staff cohort. RCs for R2* are within‐exam RC of 2.6 s^−1^ (95% CI: 2.1–3.2) and between‐exam RC of 3.2 s^−1^ (95% CI: 2.5–3.9) in the staff cohort and 3.5 s^−1^ (95% CI: 2.4–4.4) in the community cohort.

**FIGURE 7:**
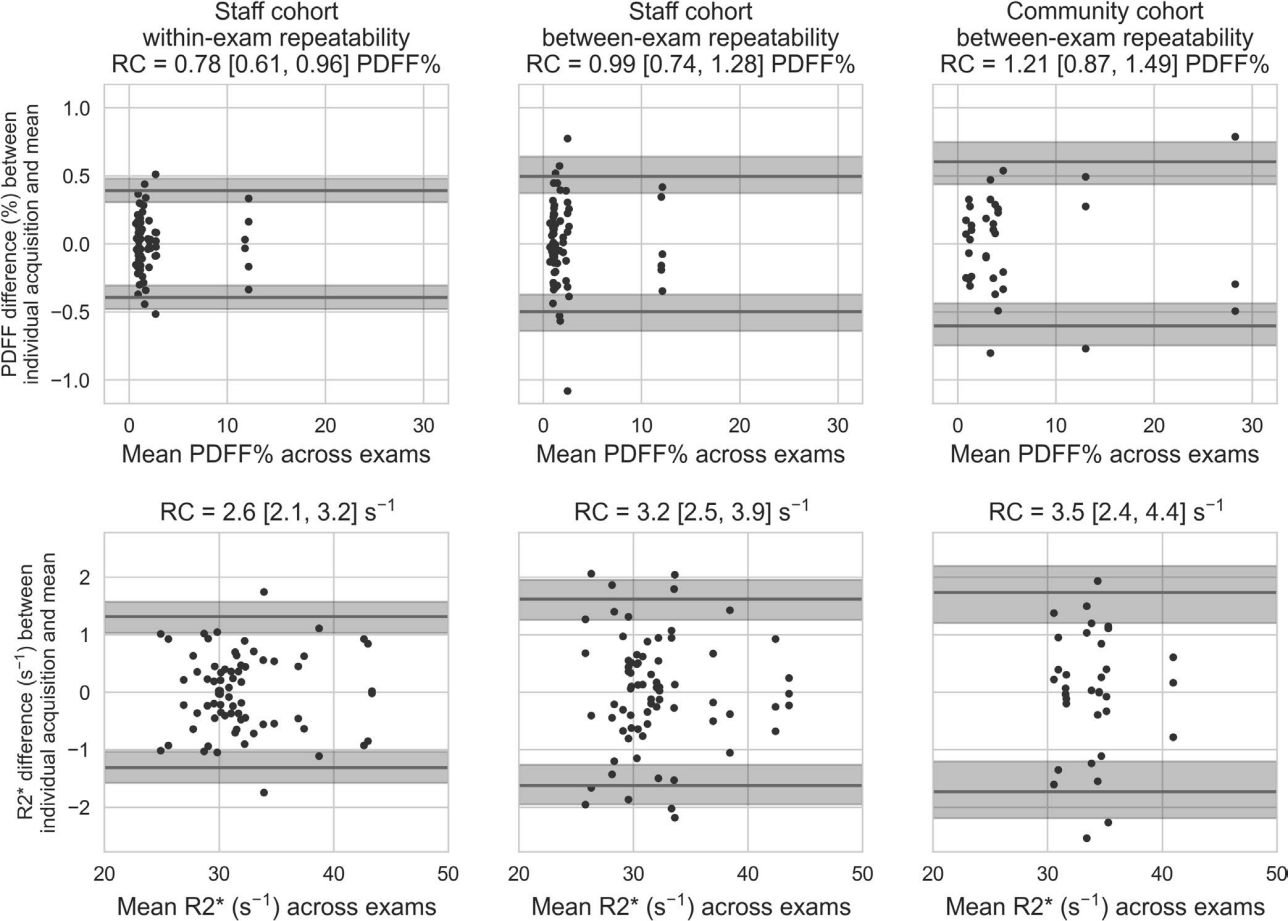
PDFF and R2* measurements were highly repeatable within and between exams using the proposed free‐breathing workflow. Shown are the PDFF and R2* repeatability analyses for all acquisitions in this work. The staff cohort was imaged twice per exam, and each participant underwent three exams, facilitating calculation of within‐exam and between‐exam repeatability. CSE‐MRI was performed once per exam for the community cohort to simulate clinical workflow. CSE‐MRI, chemical shift‐encoded MRI; PDFF, proton density fat fraction; RC, repeatability coefficient.

## 4 Discussion

This study successfully demonstrated the feasibility of combining optimized MRI suite architecture, workflow automation, and motion‐insensitive imaging to increase throughput in a focused MRI protocol for liver fat quantification. The achieved throughput of over 16 exams per hour with a focused, clinically representative protocol suggests that such protocols may be viable for high‐volume screening applications.

The two‐room smart suite design effectively decouples patient preparation from MRI room operations. Although community cohort participants required longer prep room setup times, likely reflecting lower familiarity with the MRI environment, MRI room setup times were equivalent between staff and community cohorts, suggesting that the separate prep room allows patients to prepare at a comfortable pace without impacting MRI room throughput. The consistently short and low‐variability exam times support confident scheduling of shorter exam slots, which may directly improve patient access and reduce per‐exam costs.

Although the smart suite design and workflow incur additional capital and labor costs, and a detailed cost‐effectiveness analysis is outside the scope of this work, revenue from increased patient throughput is expected to outweigh these costs. Additional capital costs for this suite design include an additional MRI room door, patient table, and additional coils. At UW Health Eastpark Medical Center, this workflow has increased throughput by approximately 50%, and an estimated 6 months were needed to recoup these additional capital costs. The workflow also requires increased staffing due to the additional table. While this study used five staff members to ensure that personnel availability did not limit throughput, fewer may be needed in routine clinical use. In current clinical practice at this site, a total of 6 or 7 staff are assigned for operation of two adjacent smart suites with a shared control room. Potential labor‐reduction strategies include assigning one technologist per table or staggering exams and sharing staff coverage across multiple suites, as would be enabled by a shared control room design. Future work will evaluate the cost‐effectiveness of the high‐staffing, high‐throughput smart suite design with that of conventional single‐door suites with fewer staff.

Although this study aimed to demonstrate high‐throughput workflow, the focused imaging protocol could also be integrated into existing clinical schedules. Clinical MRI protocols are often longer and have more variable exam lengths, creating unpredictable scheduling gaps. Focused exams using this workflow could fill such gaps, increasing daily throughput without major scheduling changes. Such an approach may also improve patient access by enabling same‐day scheduling of focused MRI exams immediately after an imaging order is placed.

The noncontrast nature of CSE‐MRI is well‐aligned with the goals of rapid, focused MRI. Although intravenous (IV) contrast is frequently administered in clinical liver MRI, it is not required for accurate diagnosis of many liver diseases, such as MASLD [15]. Contrast administration, typically used for detection and characterization of focal liver lesions, increases overall workflow time through the need for IV placement, injection, and post‐contrast acquisitions. For these reasons, avoiding the use of contrast reinforces the throughput advantages of focused protocols. When contrast‐enhanced imaging is required, the proposed architecture remains compatible. In current clinical practice at this site, IV placement occurs in dedicated preparation areas outside the MRI suite, so that prep and MRI room workflows continue to overlap.

Several aspects of this workflow may benefit from additional optimization. Granular timing analysis showed that prescan duration was comparable to the total CSE‐MRI acquisition time, presenting a potential target for reducing exam time. The autoprescription platform failures observed in this study were specific to the vendor‐provided prototype software rather than the deep‐learning network itself and may be addressed in future vendor software releases.

Other centers have also reported high‐throughput MRI suite architectures and workflows. Herold et al. [12] reported an architecture utilizing three MRI systems with dockable tables and dedicated preparation bays, reducing table turnaround times by approximately 45% compared with a reference facility. Similarly, Recht et al. [7] described an MRI suite with two doors, two tables, and a dedicated external prep room, reporting mean turnaround times of 4:32 min compared to 8:57 in conventional suites. If the focused, AI‐assisted protocol from the present work were implemented in a conventional suite, combining the mean exam time of 1:56 min of this work with the 8:57 conventional turnaround reported by Recht et al. [7] would suggest a throughput of approximately 5.5 exams per hour. This is dramatically lower than the 16.1 exams per hour observed in the smart suite and indicates that the suite architecture, rather than the focused protocol alone, accounts for much of the observed throughput gain.

Beyond architectural design, prior work has explored focused liver MRI protocols to reduce exam times. Pooler et al. [42] described a focused CSE‐MRI protocol for liver fat quantification that included a three‐plane localizer, a breath‐held 3D CSE‐MRI acquisition, and a breath‐held axial T2‐weighted SSFSE acquisition, achieving total MRI exam times of 6:48 ± 3:00 min. The proposed workflow in the present work, by comparison, achieved exam times under 3 min with substantially reduced variability (SD: 7–21 s vs. 3 min). This improvement likely reflects the use of a free‐breathing CSE‐MRI acquisition, which avoids variability introduced by breath‐hold failures and repeat acquisitions, as well as automated image prescription and a simplified protocol without T2‐weighted imaging.

In addition to workflow improvements, this work validated the performance of motion‐insensitive FAM CSE‐MRI for quantifying PDFF. The method demonstrated technical success with all exams yielding diagnostic‐quality images, with the majority rated as having very good or excellent image quality. PDFF repeatability was similar to that reported by Tang et al. [43] (RC = 1.37% absolute PDFF for FAM CSE‐MRI compared to 2.69% for conventional breath‐held CSE‐MRI). For broader context, a widely cited meta‐analysis of conventional CSE‐MRI by Yokoo et al. [44] reported test–retest RC = 2.99% absolute PDFF, though direct comparison is limited by differences in ROI‐placement methodologies across studies.

### 4.1 Limitations

The sample size was relatively small, with 12 participants per cohort, primarily due to the logistical challenges of performing a queued study in a larger cohort. However, this sample size was sufficient to evaluate the feasibility of this architecture and workflow, and repeated imaging in each participant yielded 72 total exams, enabling robust evaluation of PDFF measurement repeatability that could not be achieved with unique participants. Participants were healthy volunteers rather than patients with complex clinical conditions or mobility challenges, and workflow performance may differ in more clinically challenging populations. While no participants had a history of liver disease, recruitment for the community cohort intentionally included individuals with elevated BMI who may be at higher risk for MASLD, and this was reflected in a broader and higher range of PDFF values in this cohort. Importantly, however, the study was not designed to stratify performance by PDFF or disease state, and subgroup analyses in this cohort would not be well‐powered.

This study focused on the application of CSE‐MRI for PDFF quantification, given its clinical relevance as a biomarker for MASLD diagnosis and treatment monitoring [45]. While CSE‐MRI also provides estimates of R2*, a well‐described biomarker of liver iron overload, no participant had elevated R2* values, and future studies focusing on the iron quantification application are warranted. Further, this study did not include other MRI techniques such as MR elastography, which is often incorporated alongside CSE‐MRI in rapid liver protocols to assess MASLD‐associated liver fibrosis. Future studies should expand this approach to encompass a broader range of MRI methods, including MR elastography and contrast‐enhanced imaging, as well as diverse patient populations and pathologies.

This study was also performed at a single center using a single vendor platform, though the individual technical components, including the FAM acquisition [29, 43] and autoprescription network [21, 24], have been independently validated in large cohorts across multiple sites and vendors. Furthermore, this study assessed the workflow impact within a highly controlled, prospective design with sequential, identically protocoled exams. While dedicated single‐protocol shifts are a known strategy to improve clinical workflow [46], most clinical scheduling templates mix protocols of varying lengths. Further studies will help establish whether these results translate to a general clinical environment, particularly through multicenter, multivendor comparisons with conventional single‐door MRI suites. A direct comparison was not performed in this study, though prior studies provide context for the efficiency gains observed [7, 12]. Investigation into patient satisfaction and technologist feedback may also help engage these key stakeholders in future optimization.

### 4.2 Conclusion

This work combined optimized MRI suite architecture, workflow automation, and motion‐insensitive imaging to achieve a high‐throughput workflow for liver PDFF quantification. The proposed workflow provides a generalizable framework for efficiently performing focused MRI protocols, which may reduce cost and expand access to MRI.

## Funding

This work was supported by the National Institutes of Health (R01EB031886) and the National Science Foundation Graduate Research Fellowship Program (2137424).

## Conflicts of Interest

G.C.F., J.S., J.H., A.P., D.T.H., D.T., D.H., and S.B.R. declare no conflicts of interest. J.T. is a shareholder of GE HealthCare. C.J.B. and D.W.R. are employees of GE HealthCare. Unrelated to this work, D.H. and S.B.R. have ownership interests in Calimetrix. Additionally, S.B.R., unrelated to this work, has ownership interests in Reveal Pharmaceuticals, Elucent Medical, VistaAI, and RevOps and provides consulting services to Amgen. Further, the University of Wisconsin receives research support from GE HealthCare and Bracco Diagnostics.

## Data Availability

Data generated or analyzed during the study are available from the corresponding author upon reasonable request.
